# The Effect of Self-Assembling Peptide RADA16-I on the Growth of Human Leukemia Cells *in Vitro* and in Nude Mice

**DOI:** 10.3390/ijms10052136

**Published:** 2009-05-14

**Authors:** Chengkang Tang, Ximing Shao, Binbin Sun, Wenli Huang, Xiaojun Zhao

**Affiliations:** 1 Institute for Nanobiomedical Technology and Membrane Biology, West China Hospital, Sichuan University, Chengdu, 610041, China; E-Mails: tangck@126.com (C.T.); shaoximing07@163.com (X.S.); sunbb@163.com (B.S.); huangwenli133@163.com (W.H.); 2 West China Medical School, West China Hospital, Sichuan University, Guo Xue Xiang 37, Chengdu, 610041, Chengdu, China; 3 Center for Biomedical Engineering, NE47-379, Massachusetts Institute of Technology, Cambridge, MA 02139-4307, USA

**Keywords:** self-assembling peptide RADA16-I, nanofiber scaffolds, mimic extracellular matrix, tumor microenvironments, cancer therapy

## Abstract

Nanofiber scaffolds formed by self-assembling peptide RADA16-I have been used for the study of cell proliferation to mimic an extracellular matrix. In this study, we investigated the effect of RADA16-I on the growth of human leukemia cells *in vitro* and in nude mice. Self-assembly assessment showed that RADA16-I molecules have excellent self-assembling ability to form stable nanofibers. MTT assay displayed that RADA16-I has no cytotoxicity for leukemia cells and human umbilical vein endothelial cells (HUVECs) *in vitro*. However, RADA16-I inhibited the growth of K562 tumors in nude mice. Furthermore, we found RADA16-I inhibited vascular tube-formation by HUVECs *in vitro*. Our data suggested that nanofiber scaffolds formed by RADA16-I could change tumor microenvironments, and inhibit the growth of tumors. The study helps to encourage further design of self-assembling systems for cancer therapy.

## 1. Introduction

For a long time, many efforts have focused on the inhibition and destruction of cancer cells for cancer treatment, but unfortunately, because of drug cytotoxicity and resistance developed in cancer cells, the applications of many traditional anticancer drugs are generally accompanied with inefficiencies and severe side effects [1,2].

Recently, researchers realized that solid tumors are organ-like structures in various microenvironments; cancer cells are embedded in an extracellular matrix and nourished by a vascular network. These microenvironments not only allow interactions among cancer cells and between cancer cells and the basal membrane, but also remove waste products. Substantial studies have determined that tumor microenvironments are critical for the proliferation of tumor cells and their response to anticancer drugs [3–5]. Thus, strategies to modulate the tumor microenvironments (including the tumor vasculature) could offer additional approaches for cancer treatment.

Previous studies have discovered that self-assembling peptide systems [6–13], made from natural amino acids, can spontaneously assemble into nanofiber scaffolds, which comprise numerous nanofibers of ~ 10 nm diameter and nanopores between 5 – 200 nm [6–8]. These peptide scaffolds have three-dimensional nanofiber structures similar to the natural extracellular matrix. Consequently, they have been used for the study of cell attachment and proliferation as biomimic synthetic extracellular matrix [7,9,10].

The self-assembling peptide RADA16-I (Ac-RADARADARADARADA-CONH_2_), a simple model oligopeptide, is characterized by the regular repeats of alternating ionic hydrophilic and hydrophobic amino acids, and usually forms stable a β-sheet structure in water. RADA16-I molecules can not only spontaneously self-assemble to form stable nanofibers, but also form higher-order nanofiber scaffolds in the presence of monovalent cations or physiological media [8]. Here, we asked whether nanofiber scaffolds formed by RADA16-I could change the tumor microenvironments as biomimetic extracellular matrix, and influence the growth of tumors.

In this study, we firstly confirmed the self-assembling ability of RADA16-I by using atomic force microscopy (AFM). Secondly, MTT assay was performed to examine the effect of RADA16-I on the proliferation of human leukemia cells and human umbilical vein endothelial cells (HUVECs) *in vitro*. We next examined the inhibition of RADA16-I against human leukemia xenograft growth in nude mice. Finally, we evaluated the *in vitro* effect of RADA16-I on the vascular tube-formation by HUVECs, which are critical to form new capillaries in the vicinity of solid tumors and promote the formation of three-dimensional vascular tubes [14,15].

## 2. Results and Discussion

### 2.1. Self-assembly Assessment of RADA16-I

**Figure 1.**
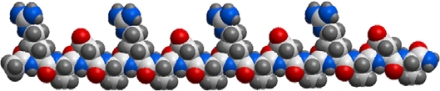
Schematic molecular model of Peptide RADA16-I. RADA16-I has regular repeats of ionic hydrophilic and hydrophobic amino acids, and forms stable β-sheet structure in aqueous solution with two distinct surfaces — hydrophilic surface and hydrophobic surface. All the hydrophobic alanine residues form the hydrophobic surface, arginine and aspartic acid residues constitute the hydrophilic surface with regular repeats. The dimensions of the RADA16-I molecule are about 5 nm in length, 1.3 nm in height, and 0.8 nm in width [16].

The self-assembling peptide RADA16-I (Figure 1) is a self-complementary peptide, which forms stable β-sheet structure in aqueous solution with two distinct surfaces—one hydrophilic, the other hydrophobic. All the hydrophobic alanine residues shield themselves from water and self-assemble on the hydrophobic surface, on the other hand, arginine and aspartic acid residues can form complementary ionic bonds with regular repeats on the hydrophilic surface. Based on these intermolecular forces, RADA16-I molecules can spontaneously self-assemble to form stable nanofibers and higher-order nanofiber scaffolds in physiological media.

**Figure 2.**
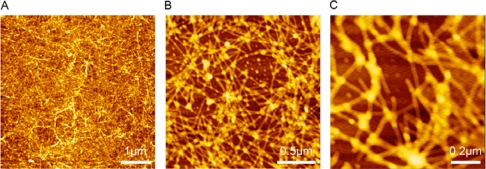
AFM images of RADA16-I. The working solution of RADA16-I was prepared at 100 μM concentration by using Milli-Q water. The nanostructure of RADA16-I was scanned at different scales, 5 × 5 μm^2^ (A), 2 × 2 μm^2^ (B) and 1 × 1 μm^2^ (C). Dense nanofibers were observed, indicating peptide RADA16-I has excellent self-assembling ability to form nanofiber scaffolds.

In this study, the nanostructure of RADA16-I was observed by using AFM (Tapping Mode) at different scales (5 × 5 μm^2^, 2 × 2 μm^2^ and 1 × 1 μm^2^). As shown in Figure 2, dense nanofibers formed by RADA16-I were observed, a result consistent with a previous study [17]. The result confirms that peptide RADA16-I has excellent self-assembling ability to form nanofibers and higher-order nanofiber scaffolds.

### 2.2. Cytotoxicity Assays in vitro

**Figure 3.**
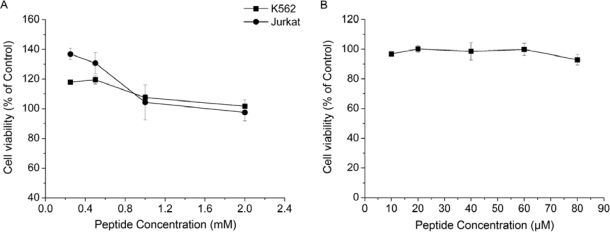
Cytotoxicity of RADA16-I against human leukemia cells and HUVECs by MTT proliferation assay. (A) MTT assay showed that RADA16-I had no cytotoxicity for both K562 and Jurkat cells. After treated with RADA16-I solution at the maximal concentration (2mM), the relative viabilities of K562 and Jurkat cells were 101.66 ± 4.25% and 97.44 ± 5.66%, respectively. RADA16-I solutions at lower concentrations even enhanced the proliferation of cells. (B) MTT assay displayed that RADA16-I had almost no cytotoxicity against HUVECs even at 80 μM peptide concentration (92.90 ± 3.54% viability). Data shown are Means ± SD.

Previous studies have shown that nanofiber scaffolds formed by self-assembling peptides have no cytotoxicity, and support the attachment and growth of a variety of mammalian primary cells in tissue culture [6–8]. Similarly, RADA16-I is nontoxic and supports not only the growth of cells [8], but also tissue regeneration [18]. The results of a MTT assay are consistent with early studies, showing that RADA16-I had no cytotoxicity for both K562 and Jurkat cells even at 2 mM peptide concentration; the relative viabilities were 101.66 ± 4.25% for K562 cells and 97.44 ± 5.66% for Jurkat cells (Figure 3A). RADA16-I even promoted the growth of leukemia cells at lower peptide concentrations (Figure 3A). Moreover, MTT assay also showed that RADA16-I had no cytotoxicity against HUVECs, there was almost no reduction in HUVEC viability (90 – 100% viability) after treatment with peptide RADA16-I at varying concentrations ((10 – 80μM) (Figure 3B). Taken together, the results of MTT assay suggested that peptide RADA16-I had an excellent biocompatibility without obvious cytotoxicity as described [8,18].

### 2.3. Inhibition of RADA16-I on K562 Tumor Growth in vivo

**Figure 4.**
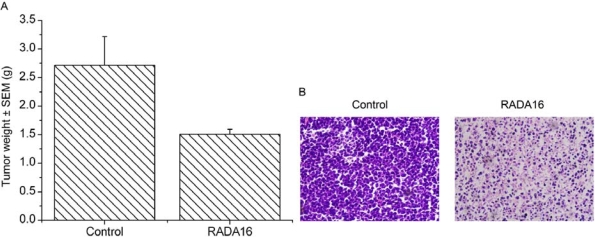
Effect of RADA16-I on K562 tumor growth. We analyzed the effect of RADA16-I on K562 tumor growth by using mouse models. (A) After treatment with RADA16-I, average tumor weight in treatment group (1.51 ± 0.08 g) was markedly decreased as compared with that in control group (2.72 ± 0.50 g), data shown are Means ± SEM, P < 0.05. (B) Histological examination of tumors. Tumors were fixed with formaldehyde, and sectioned for histological examination by H & E staining. H & E staining showed that massive cell death existed in tumors treated by RADA16-I compared with the controls. These results suggested that RADA16-I could inhibit the K562 tumor growth *in vivo*.

After examining the cytotoxicity of RADA16-I against leukemia cells *in vitro*, we further analyzed the effect of RADA16-I on the growth of K562 tumors in nude mice. After the sixth treatment, we found that the average tumor weight (1.51 ± 0.08 g) in RADA16-I treatment group had been markedly decreased, compared with that (2.72 ± 0.50 g) in a control group (Figure 4A). Other than these effects, animals treated with RADA16-I were normal and did not show any sign of physical weakness throughout the experiment. Finally, tumors were fixed with formaldehyde, and sectioned for histological examination by H&E staining. As shown in Figure 4B, H&E staining showed that mass proliferation existed in tumor cells from control mice, whereas the structural pattern of tumors treated with RADA16-I was replaced by massive cell death in which the cells were characterized by acidophilic homogeneous masses without nuclei. These data suggested that RADA16-I could inhibit the K562 tumor growth *in vivo* though peptide has no cytotoxicity against cancer cells *in vitro*.

### 2.4. Inhibition of Vascular Tube-formation by RADA16-I

**Figure 5.**
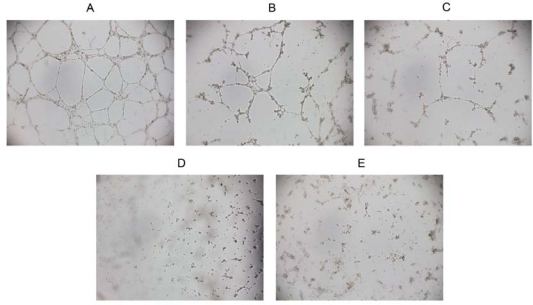
Inhibition of vascular tube-formation *in vitro*. We evaluated the effect of RADA16-I on the tubes formation by HUVECs *in vitro*. (A) HUVECs treated with control solution can migrate to form a well-arranged network of tubes within 9 h. After treated with 20 μM (B) or 40 μM (C) RADA16-I, the network-like structures were broken. Treatment with 60 μM (D) or 80 μM (E) RADA16-I completely inhibited the tube formation. These data suggested that RADA16-I could inhibit the formation of vascular tubes.

The formation of new capillaries by HUVEC is critical for the migration of endothelial cells and the formation of neovascularization [14]. In our study, we evaluated the effect of RADA16-I on tube formation by HUVECs. As seen in Figure 5, HUVECs without peptide treatment migrated to form a well-arranged network of tubes within 9 h, while this effect was significantly decreased in the presence of RADA16-I. After treated with 20 μM or 40 μM RADA16-I, the endothelial network-like structures were broken. Furthermore, treatment with 60 μM or 80 μM RADA16-I yielded maximal inhibition with the almost complete absence of tube formation. However, MTT assay showed that RADA16-I is nontoxic to HUVECs even at 80 μM peptide concentration (Figure 3B), suggesting the inhibition of vascular tube-formation is not caused by cell death or cytotoxicity. Taken together, these results indicated that RADA16-I could inhibit the formation of neovascularization without cytotoxic effect, and consequently lead to prevention of tumor growth.

## 3. Experimental Section

### 3.1. Preparation of Peptide RADA16-I

The peptide RADA16-I (PuraMatrix^TM^, Ac-RADARADARADARADA-CONH_2_) was purchased from BD Bioscience (Bedford, MA, USA). The working solutions of RADA16-I were prepared at different concentrations with sterile water (18 MΩ; Millipore Milli-Q system) and stored at 4 °C.

### 3.2. Cell Cultures

Human umbilical vein endothelial cells (HUVECs) were isolated from human umbilical cord tissue by collagenase digestion as described previously [19]. The harvested cells were plated on gelatin-coated culture bottles in medium 199 containing 20% fetal calf serum, basic fibroblast growth factor (bFGF, 10 ng/mL), vascular endothelial growth factor (VEGF, 2 ng/mL), 200 U/mL penicillin and 200 μg/mL streptomycin. After 3 – 4 passages, HUVECs were collected to use. Two human leukemia cell lines, K562 and Jurkat, were grown in RPMI 1640 medium supplemented with 10% fetal calf serum and antibiotics (100 units/mL penicillin G, 100 μg/mL streptomycin). All cell lines were incubated at 37 °C in a 5% CO_2_ humidified atmosphere.

### 3.3. Self-Assembly Assessment of RADA16-I with Atomic Force Microscopy

RADA16-I working solution was prepared at a concentration of 100 μM with Milli-Q water. Approximately 5 μL of RADA16-I solution was evenly deposited onto a freshly cleaved mica surface at room temperature. After 30 seconds, the mica surface was rinsed with 1,000 μL of Milli-Q water to remove unattached peptides. Sample on the mica surface was then air-dried and scanned by Atomic Force Microscopy (AFM) in air.

AFM images were collected by using the tapping mode on a SPI4,000 Probe Station & SPA-400 SPM Unit (Seiko Instruments Inc., Chiba, Japan). All images were acquired by utilizing a 20 μm Scanner (400) and an Olympus Si-DF20 microcantilever as well as tip (Si, tip radius 10 nm, rectangular base 200.00 μm) with the spring constant of 12.00 N/m. The cantilever’s free resonance frequency is 127.00 KHz. To show the nanostructure formed by self-assembling peptide RADA16-I, AFM images were scanned and collected at 5 × 5 μm^2^, 2 × 2 μm^2^ and 1 × 1 μm^2^ scales.

### 3.4. MTT Proliferation Assay

The effect of RADA16-I on the viability of human leukemia cells and HUVECs *in vitro* was assessed by a MTT assay [20]. Briefly, cells in 100 μL of fresh medium were seeded onto 96-well plates at a density of 5 × 10^3^ cells/well for K562, 2 × 10^4^ cells/well for Jurkat and 1 × 10^4^ cells/well for HUVECs, respectively. After 24 h incubation at 37 °C, 10 μL of RADA16-I solution and control solution (sterile water) were added and incubated for 48 h. Thereafter, 20 μL of MTT reaction solution (5 mg/mL MTT in PBS; Sigma) was added to each well, and the plates were incubated at 37 °C for 4 h. 10% SDS solution (100 μL) containing 0.01 M HCL was immediately added to each well to solubilize deposit and the plates were incubated overnight. Spectrometric absorbance was measured at 570 nm by a μQuant microplate reader (Bio-Tek). Cell viability was determined relative to the control.

### 3.5. Inhibition of RADA16-I against K562 Tumor Growth in Mice

BALB/c nude mice (4–6 weeks) were obtained from the Experimental Animal Center, Sichuan University. Approximately 1 × 107 K562 cells suspended in 200 μL of serum-free culture medium RPMI 1,640/Matrigel mixture (1:1) were injected s.c. into the right flank of nude mice. Four days after implantation, solid tumor formed. The mice were then randomly assigned into two groups, RADA16-I treatment group and control group. Each group contained seven mice aged 5 – 6 weeks. In treatment group, RADA16-I solution (2 mM, 0.1 mL) was injected to the brim of the tumors every 3 days. Parallel experiments were performed in control group, in which 0.1 mL of sterile water without peptide was injected in the same manner. Mice were sacrificed 18 days after the first treatment to measure tumor masses and to perform histological examination by H & E staining.

### 3.6. Vascular Tube Formation Assay in vitro

Vascular tube formation assay *in vitro* was performed according to the previous description [14,15]. Briefly, 96-well plates were coated with Matrigel (0.05 mL) and incubated at 37 °C for 1 h to promote gelling. HUVECs suspension (1.4 × 10^4^ cells/well) were seeded on the Matrigel and treated with RADA16-I solutions at various concentrations for 9 h. Phase-contrast microscope (Olympus IX71, Tokyo, Japan) was used to record tube formation by HUVECs.

### 3.7. Statistical Analysis

Statistical analysis was performed by using the SPSS 13.0 for windows package, and student’s t test was used to determine statistical significance.

## 4. Conclusions

We have investigated the self-assembling ability of peptide RADA16-I and its effect on the growth of human leukemia cells *in vitro* and *in vivo*. AFM images showed that peptide RADA16-I molecules have excellent self-assembling ability to form nanofiber scaffolds, which have been used as a biomimetic extracellular matrix. Most importantly, the study demonstrated that RADA16-I molecules inhibit the growth of K562 tumors in nude mice though they have no cytotoxicity against cancer cells *in vitro*. Furthermore, we found that RADA16-I inhibited the vascular tube-formation by HUVECs without cytotoxic effect *in vitro*. Taken together, our data indicated that RADA16-I molecules self-assemble into nanofiber scaffolds, which could change tumor microenvironments (including vascular tubes), and inhibit the growth of tumors. This study reveals the feasibility of designing novel nanomaterials based on self-assembling systems for cancer treatment.
